# Editorial: Advanced nanotechnology for integrated diagnosis and treatment

**DOI:** 10.3389/fbioe.2026.1891607

**Published:** 2026-07-16

**Authors:** Liming Zhu, Ye Zhao, Xiaolong Li

**Affiliations:** 1 School of Basic Medicine Sciences, Anhui Medical University, Hefei, Anhui, China; 2 School of Materials Science and Engineering and Key Laboratory of Magnetic Molecules and Magnetic Information Materials of Ministry of Education, and Shanxi Key Laboratory of Advanced Magnetic Materials and Devices, Shanxi Normal University, Taiyuan, China

**Keywords:** advanced nanotechnology, clinical data, disease diagnosis, nanoenzyme, therapeutic monitoring

Advanced nanotechnology is increasingly contributing to integrated strategies for disease diagnosis, treatment, and therapeutic monitoring. In conventional biomedical approaches, diagnosis and intervention are often performed as separate procedures. By contrast, nanotechnology-enabled platforms offer opportunities to integrate molecular recognition, biomarker detection, imaging guidance, targeted delivery, and dynamic therapeutic regulation within coordinated systems. The conceptual shift is particularly relevant to complex diseases, including cancer, vascular disorders, infectious diseases, and organ dysfunction. In these settings, early detection, precise localization, individualized intervention, and real-time assessment of therapeutic responses may help improve clinical decision-making and therapeutic outcomes. Against this background, the Research Topic “Advanced nanotechnology for integrated diagnosis and treatment” was launched to highlight recent progress in this interdisciplinary field.

Several contributions in this Research Topic first highlight diagnostic precision as an important basis for integrated diagnosis and treatment, spanning both clinical lesion localization and molecular signal detection. “Zhao et al.” showed that VBN-guided biopsy was associated with higher diagnostic accuracy, shorter procedure time, and fewer complications in selected small and near-pleural nodules. This finding highlights that accurate localization and safe sampling are prerequisites for subsequent therapeutic decision-making. Reliable lesion identification remains important for maximizing the clinical value of subsequent nanotheranostic strategies.

Beyond anatomical localization, several studies further extend diagnostic precision to molecular recognition and dynamic biosensing. “Luo et al.” reviewed nanozyme-assisted CRISPR diagnostic systems, in which nanozymes are integrated with CRISPR/Cas platforms to enable cascade signal amplification and visual readout. This strategy helps address key limitations of conventional CRISPR diagnostics, including weak signal output, dependence on natural enzymes, and the need for specialized equipment.

While nanozyme-assisted CRISPR systems emphasize signal amplification and visual readout, the two HSA biosensor studies “Chen et al.” and “Chen et al.”move this diagnostic logic further toward label-free detection and dynamic regulation. By using Dirac semimetal heterostructures and optical topological edge states, respectively, these studies reported highly sensitive, label-free detection and suggested the potential for external-voltage modulation of nanozyme-related responses. Together, these studies suggest that the integration of diagnosis and treatment depends not only on improved detection sensitivity, but also on sustained diagnostic capabilities. Such capabilities may help connect spatial localization, molecular recognition, and dynamic feedback within coordinated therapeutic systems.This progression provides a useful perspective on how future diagnostic platforms may become more sensitive, feedback-capable, and adaptable.

With improved diagnostic precision as a foundation, therapeutic nanoplatforms are increasingly being designed to integrate imaging guidance, multimodal intervention, and biomimetic regulation. Using hepatocellular carcinoma as an example, “Shen et al.” summarized the application of nanotheranostic platforms in multimodal imaging and combined therapies, including photothermal, photodynamic, and immunotherapy. These advances highlight the potential of nanotechnology to support more precise intervention in complex tumor microenvironments.

While these disease-oriented nanotheranostic strategies are promising, they also raise a broader translational question: how can increasingly complex nanoplatforms be made safe, controllable, and manufacturable? To address this challenge, the article “Chen et al.” proposed the concept of the “minimal platelet interface” (MPI) and related gating strategies. These ideas provide a useful framework for considering how biomimetic therapeutic systems may move from functional validation toward controllable design, standardized manufacturing, and regulatory evaluation.

Beyond controllable and manufacturable design, the contribution on surgical nanorobots “Guo et al.” further points to the emerging role of active execution in integrated nanomedicine.By integrating multimodal imaging, artificial intelligence, and closed-loop control, micro/nanorobots may enable more active forms of navigation, localized intervention, and therapeutic feedback.Together, these studies suggest that therapeutic nanoplatforms are moving beyond functional integration toward systems that are safer, more controllable, more reproducible, and more closely aligned with clinical translation.They also point to the emerging role of active navigation and feedback-controlled intervention in future nanomedicine.

Collectively, these contributions highlight the progression of advanced nanotechnology from precise diagnosis and molecular sensing to therapeutic integration, biomimetic control, and active intervention. By linking clinical localization, nanozyme-assisted CRISPR diagnostics, dynamically tunable biosensors, cancer nanotheranostics, platelet-mimicking systems, and nanorobots, this Research Topic illustrates a possible path toward more sensitive, controllable and clinically translatable diagnostic and therapeutic platforms.Nevertheless, challenges remain in biosafety, immune compatibility, manufacturing scalability, standardization, and regulatory evaluation. A common message across these studies is that successful clinical translation is likely to depend not only on functional innovation, but also on controllable design, reproducible production, consistent characterization, and disease-specific validation.We hope that this collection provides readers with an overview of recent advances and inspires further development of safer, more adaptive, and clinically translatable nanomedicine systems.

